# Single-domain antibodies against SARS-CoV-2 RBD from a two-stage phage screening of universal and focused synthetic libraries

**DOI:** 10.1186/s12879-024-09022-8

**Published:** 2024-02-13

**Authors:** Fangfang Chen, Zhihong Liu, Wei Kang, Fan Jiang, Xixiao Yang, Feng Yin, Ziyuan Zhou, Zigang Li

**Affiliations:** 1 Department of Pharmacy, Shenzhen Hospital, Southern Medical University, Shenzhen, China; 2 Pingshan Translational Medicine Center, Shenzhen Bay Laboratory, Shenzhen, China; 3 NanoAI Biotech Co., Ltd, Pingshan District, Shenzhen, China; 4 State Key Laboratory of Chemical Oncogenomics, School of Chemical Biology and Biotechnology, Peking University Shenzhen Graduate School, Shenzhen, China; 5 National Cancer Center, National Clinical Research Center for Cancer/Cancer Hospital & Shenzhen Hospital, Chinese Academy of Medical Sciences and Peking Union Medical College, Shenzhen, China

**Keywords:** SARS-CoV-2, RBD, Single-domain antibody, Synthetic library, Phage display

## Abstract

**Background:**

Coronavirus disease 2019 (COVID-19) is an evolving global pandemic, and nanobodies, as well as other single-domain antibodies (sdAbs), have been recognized as a potential diagnostic and therapeutic tool for infectious diseases. High-throughput screening techniques such as phage display have been developed as an alternative to in vivo immunization for the discovery of antibody-like target-specific binders.

**Methods:**

We designed and constructed a highly diverse synthetic phage library sdAb-U (single-domain Antibody - Universal library ) based on a human framework. The SARS-CoV-2 receptor-binding domain (RBD) was expressed and purified. The universal library sdAb-U was panned against the RBD protein target for two rounds, followed by monoclonal phage ELISA (enzyme-linked immunosorbent assay) to identify RBD-specific binders (the first stage). High-affinity binders were sequenced and the obtained CDR1 and CDR2 sequences were combined with fully randomized CDR3 to construct a targeted (focused) phage library sdAb-RBD, for subsequent second-stage phage panning (also two rounds) and screening. Then, sequences with high single-to-background ratios in phage ELISA were selected for expression. The binding affinities of sdAbs to RBD were measured by an ELISA-based method. In addition, we conducted competition ELISA (using ACE2 ectodomain S19-D615) and SARS-CoV-2 pseudovirus neutralization assays for the high-affinity RBD-binding sdAb39.

**Results:**

Significant enrichments were observed in both the first-stage (universal library) and the second-stage (focused library) phage panning. Five RBD-specific binders were identified in the first stage with high ELISA signal-to-background ratios. In the second stage, we observed a much higher possibility of finding RBD-specific clones in phage ELISA. Among 45 selected RBD-positive sequences, we found eight sdAbs can be well expressed, and five of them show high-affinity to RBD (EC_50_ < 100nM). We finally found that sdAb39 (EC_50_ ~ 4nM) can compete with ACE2 for binding to RBD.

**Conclusion:**

Overall, this two-stage strategy of synthetic phage display libraries enables rapid selection of SARS-CoV-2 RBD sdAb with potential therapeutic activity, and this two-stage strategy can potentially be used for rapid discovery of sdAbs against other targets.

**Supplementary Information:**

The online version contains supplementary material available at 10.1186/s12879-024-09022-8.

## Background

Since December 2019, a novel and highly transmissible severe acute respiratory syndrome coronavirus 2 (SARS-CoV-2, COVID-19) [1, 2] has erupted on a large scale worldwide and spread rapidly. As of May 2023, more than 750 million people have been infected and about 7 million lives have been claimed. These numbers are still rising. The global COVID-19 pandemic poses serious challenges to patients, healthcare systems, and economic and social activity. Although the SARS-CoV-2 vaccine is widely used around the world, the vaccine’s protective effect is greatly reduced in people with weakened immunity system, such as the elderly or people with immune-compromised conditions. Vaccine alone is not enough to end the pandemic [3]. The high transmissibility, rapid variations of viral sequences, and potential for immune escape of the SARS-CoV-2 variants further underscores the need for rapid development of antibodies or other affinity reagents for the diagnostics, prevention, and treatment of SARS-CoV-2. Indeed, monoclonal antibodies (mAb) have had tremendous success in treating a variety of diseases, and several mAb have been approved for the treatment of COVID-19 [4–9]. However, mutations in SARS-CoV-2 have resulted in reduced sensitivity to some of the developed anti-SARS-CoV-2 mAbs [10, 11], In addition, the high production costs, large doses needed, and low-temperature requirements for transportation and storage associated with traditional mAbs make it challenging to be cost-effective for large scale applications [12].

mAb are heterogeneous macromolecules that require variable regions of heavy and light chains to bind together as antigen recognition pockets. Interestingly, antibodies with only heavy chains (HCAb) have been found to exist in camelids, which require only a single antigen-binding domain (heavy-chain variable region, VHH) to fulfill the function of an antibody [13]. The VHHs from camelids are much smaller (molecular weights of ~ 15 kD) than traditional mAbs (~ 150 kD), and have been commonly termed nanobodies (Nbs) [14, 15]. Due to their minimal size, they are particularly suited to reach hidden epitopes such as crevices of target proteins [16]. Nbs have shown great potential in biomedical applications, including cancer, infection, inflammation, and other diseases [17–19]. The first Nb has been approved to treat acquired thrombotic thrombocytopenic purpura (aTTP) in 2018 [20]. In addition, Nbs can be easily bioengineered into novel bivalent / multivalent / multispecific and high-affinity molecules [21, 22], and they can be aerosolized for direct delivery to the lungs. Howerver, the camelid origin of Nbs limits their application as human therapeutics. To reduce the risk of immunogenicity, humanizations of camel nanobodies have emerged in recent years [23, 24]. These research efforts have led to the study of single-domain antibodies (sdAb) from a variety of sources, including human germline immunoglobulin heavy chain variable (IGHV) region [25]. For biomedical applications, human-derived sdAb may be preferable because of their limited immunogenicity in patients. sdAb have several important advantages over traditional mAbs. Like Nb, sdAb can be expressed in prokaryotic systems with lower production costs, and it provides opportunities for rapid production of antiviral drugs.

The interaction between the SARS-CoV-2 and the host cell is mediated by the receptor binding domain (RBD) of the S1 subunit in its surface Spike (S) glycoprotein. The SARS-CoV-2 RBD binds to the peptidase domain (PD) of Angiotensin-converting enzyme 2 (ACE 2) [26]. Therefore, the RBD is a key region for SARS-CoV-2 binding to the ACE2 receptor, and is considered to be the most effective target for anti-SARS-CoV-2 neutralizing antibodies to date [5, 7, 8]. At present, A variety of anti-SARS-CoV-2 nanobodies targeting RBD have been reported, such as TY1 [27], WNb7 [28], and PiN-21 [29], among others. They can be flexibly constructed into multiple versions such as dimeric, trimeric, decameric, and other multivalent antibodies, which can be administered via the respiratory route and inhibit SARS-CoV-2 infection in mice in both pre-exposure and post-exposure prophylaxis environments. These SARS-CoV-2 nanobodies are mainly obtained through the “in vivo” immunization method [29–34]. The recombinant S glycoprotein or RBD protein was used to immunize camelid animals such as camels or alpacas. However, “in vivo” screening methods require a long development period (usually > 3 months) from antigen to final specific nanobodies. Therefore, rapid and efficient “in vitro” selection has become a very promising approach for obtaining single-domain antibodies, by combining high-throughput selection techniques such as phage display [35, 36] with synthetic sdAb libraries [24, 37, 38].

Synthetic libraries use gene synthesis methods to introduce random DNA sequences at CDR loci [37]. The synthetic sdAb phage library is a fully synthetic non-immune library, so it is not dependent on animal experiments, is not limited by the natural immunogenicity or toxicity of the antigen, and allows for the development and adaptation of choices without ethical considerations. In addition, since all steps are performed in vitro, conditions can be tightly controlled. This enables the development of robust differential selection and the recognition of conformation-specific antibodies. In addition, since natural antibody selection requires animal immunization, very conserved or toxic antigens should be avoided and there is usually only limited control over the immune response. In contrast, synthetic libraries do not require animal sacrifice and provide a higher diversity of binders even for highly conserved antigens in mammals, but high specificity and affinity are usually achieved when selecting from very large functional libraries [24]. There are only a few reports of the use of synthetic phage libraries to generate SARS-CoV-2 antibodies with therapeutically desired characteristics.

Here, we designed and synthesized a highly diverse phage library of sdAb with human framework (sdAb-Universal, sdAb-U), and screened it to obtain sdAbs specifically binding to RBD. The CDR1 and CDR2 of the obtained lead sequences were then assembled with random CDR3 to construct a focused (targeted) library, sdAb-RBD. By this two-stage method, high-affinity RBD-specific sdAbs can be selected within about two weeks, which is considerably faster than the “in vivo” method. Finally, we screened and obtained several sdAbs that bind to RBD with high affinities. One of the important purposes of conducting this research is to verify the feasibility of using our new synthetic sdAb library to obtain antigen-specific binders. Overall, we have established an efficient two-stage approach that reduces the dependence on large library capacity for synthetic library screening and can rapidly develop human sdAbs targeting SARS-CoV-2 RBD. In the future, our approach may also help researchers to create their synthetic libraries and to obtain new single-domain antibodies against other targets, with high efficiency.

## Methods

### Design of the universal sdAb library sdAb-U

**Fig. 1:**
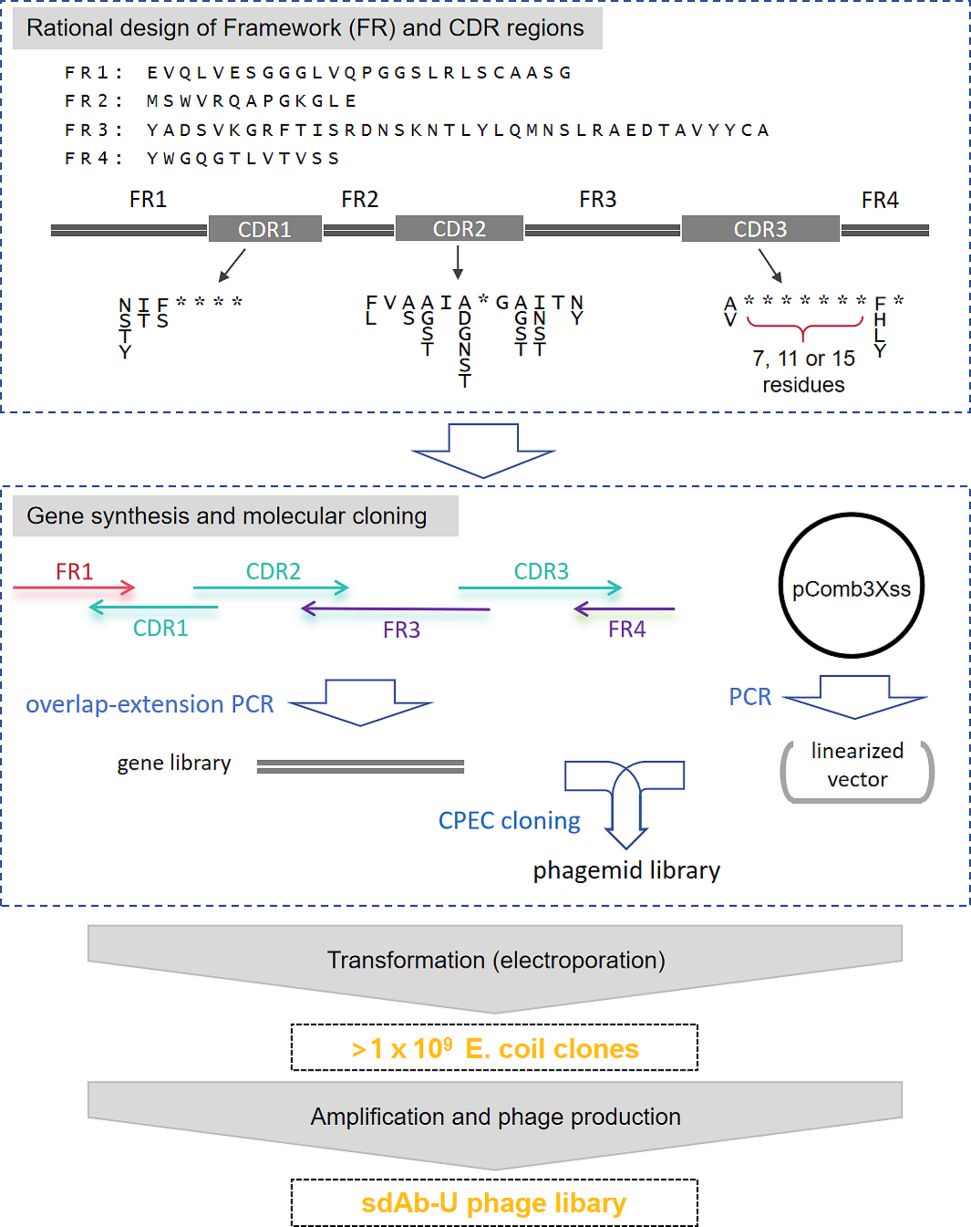
sdAb-U library construction pipeline

We used a sdAb framework (Fig. 1) based on a soluble human germline immunoglobulin heavy-chain variable region (IGHV3-66*01), which has been shown to be an ideal alternative to camelid Nbs [36]. For highly variable complementary-determining regions (CDRs), the sequence library (Fig. 1) was designed based on the work of McMahon et al. [37], which agrees well with the bioinformatics analysis of Nb sequences (comparative analysis Nb seq logo) [39]. It is observed that Nbs CDR3 varies greatly in length, and contributes most to antigen-binding affinity and specificity [13]. Therefore, three different lengths (10, 14, and 18) were chosen for CDR3. We use NNC and NNK codons for fully randomized amino acids. For variable positions not fully randomized (such as the first amino acid in the CDR1 region), we designed degenerate codons using a web-based tool SwiftLib [40]. The designed DNA sequence is further optimized for *E. coli* expression based on codon usage.

### Construction of the synthetic sdAb-U library

Three sub-libraries were constructed for the three different CDR3 lengths respectively, and then combined in subsequent phage panning. As shown in Fig. 1, the full-length sdAb DNA fragments (as a gene library) were obtained by one-pot overlap-extension PCR (OE-PCR) [41] using oligonucleotides (with degenerated codons) purchased from external primer synthesis services. PCR was performed using high-fidelity DNA polymerase (Phusion Green DNA polymerase, Thermofisher) for 20 ~ 32 cycles (with annealing temperature chosen according to Thermofisher’s online *T*_*m*_ calculator). The full-length sdAb sequences were cloned into the pComb3Xss phagemid (NBbiolab, China) using the circular polymerase extension cloning (CEPC) method [42–44]. PCR primers for linearizing the pComb3Xss vector were designed and synthesized for PCR amplification to linearize the phagemid pComb3Xss. In the final CPEC assembly and cloning reaction, the prepared PCR-linearized vector pComb3Xss and the full-length sdAb DNA fragments (molar ratio of vector to insert fragments is 1:1.2). The recombinant phagemid was electro-transformed (Bio-Rad MicroPulser electroporator) into *E. coli* TG1 bacteria at 2.5 kV (0.2 cm cuvette) and ~ 5.2ms time constant. Pre-warmed SOC medium was added and incubated at 37℃ with shaking at 250 rpm for 45 min. Finally, > 1x10^9^ bacteria clones were obtained for each sub-library.

To prepare the phage library, the cultures were inoculated to 2×TY medium with 75 mg/mL carbenicillin and 1.5% (w/v) glucose, and incubated at 37℃ with constant shaking. When the culture reached OD_600_ = 0.5, TG1 cells were infected with M13KO7 helper phages (NBbiolab, China), and incubated without shaking for 45 min at 37℃. The TG1 were harvested and resuspended in 2×TY medium with carbenicillin (75 mg/mL) and kanamycin (15 mg/mL), and cultured overnight at 30℃ with constant shaking. The next day, the cultures were centrifuged and phages were precipitated from the supernatant by adding PEG-NaCl (final concentration: 4% PEG8000, 0.5 M NaCl). After centrifugation, the precipitated phages were resuspended in sterile PBS buffer. In this way, we generated three highly diverse phage sub-libraries, and they together formed our synthetic sdAb-U library (single-domain Antibody Universal library) (Fig. 1). In addition, the focused library sdAb-RBD was constructed using the same gene library synthesis and cloning protocols as for the universal library sdAb-U.

### sdAb screening from phage library

96-well plates (Corning, high binding surface) were coated with 100 µl of 100 µg/ml purified protein (RBD or BSA) for 2 h at room temperature (RT), and blocked with PBS buffer containing 2% milk powder (w/v) for 1 h at RT. Phages library were incubated with immobilized antigen for 1 h and then washed with PBST (PBS buffer supplemented with 0.5% Tween 20). To avoid the enrichment of non-specific binders to plastic plates during panning, we first incubate the phage libraries with empty plates wells to eliminate phages that can bind to the plate surface without the immobilized target. Bound phages were eluted with 100 µl of 20 µg/ml trypsin, and were used to infect TG1 bacteria culture (OD_600_ = 0.2 ~ 0.8) at 37 °C for 45 min. The eluted phage library was amplified according to the protocol described in the above section. The antigen-specific binding of the phages library after each round of panning was assessed by polyclonal phage ELISA. Single-clone phage ELISA was also carried out using colonies on phage titration plates.

### Enzyme-linked Immunosorbent Assay (ELISA)

The entire ELISA procedure was carried out at room temperature. 96-well plates (Corning #3690) were coated with 100 µl of 100 µg/ml purified protein (RBD or BSA) for 2 h, and blocked with PBS buffer containing 2% milk powder (w/v) for 1 h. For polyclonal phage ELISA, phages from each round of panning were incubated with immobilized antigen, and bound phages were detected with anti-M13-horseradish peroxidase (HRP) polyclonal antibody (Thermofisher, MA5-29950). For the purified antibody binding assay, serially diluted sdAb (with HA-tag) solutions were added and incubated for 1.5 h, and bound sdAb were detected with monoclonal anti-HA-HRP antibody. The enzyme activity was measured with the subsequent addition of substrate EL-TMB and signal reading was carried out at 450 nm using a Microplate Spectrophotometer.

### Protein expression and purification

The gene sequences of the sdAb were amplified with PCR, TY1 nanobody DNA sequence were synthesized and subcloned into a pET-21(a+) expression vector, which contains a C-terminal 6xHis + HA tag. The expression construct was transformed into a BL21(DE3) chemically competent *E. coli* for protein expression.

The overnight culture with the selected colony was inoculated in 1 L LB media with the correct antibiotics. The temperature was decreased to 18℃when OD_600_ of culture reached 0.6, and the recombinant sdAb protein expressing was induced overnight with 0.5mM IPTG. Bacterial was harvested and resuspended in lysis buffer (50mM PBS, 2mM PMSF, pH 7.4). Protein was purified with Ni column (HiTrap Excel, GE Healthcare) and gel filtration (Superdex S75 column, GE Healthcare).

RBD (R319-F541) and human ACE2 ectodomain(S19-D615) protein were expressed with the Bac-to-Bac Baculovirus Expression System (Invitrogen). The corresponding gene of two proteins were subcloned into a modified pFastBac1 vector (Invitrogen), which contains a N-terminal GP67 secreting signal peptide sequence and a C-terminal 6xHis purification tag. The expressing construct was transformed into the bacterial DH10bac competent cells, the recombinant bacmid was extracted and transfected into the sf9 insect cells with the Cellfectin II reagent (Invitrogen). After two rounds of amplification, the recombinant baculovirus with high titer were harvested and mixed with Hi5 insect cell (2 × 10^6^ cells per mL). After 60 h of infection, the cell culture containing the secreted proteins was harvested. Protein purified with Ni-column (HiTrap Excel, GE Healthcare) and gel filtration column (Superdex 200, GE Healthcare); PBS buffer was used for all purification steps.

### Competition ELISA for sdAb39 and ACE2 binding to SARS-CoV-2 RBDs

Each well of a 96-well plate was coated with 100 µL of 20 µg/mL RBD solution, and the control was coated with 20 µg/mL BSA protein solution, placed on a shaker, minimum speed, 60 min. The coated solution was discarded, and 300 µL of PBS-T was washed 3 times, and 300 µL of fresh 2% Milk-PBS-T (the blocking solution) was added to each well, RT, minimum speed, 45 min. sdAb39, TY1, and ACE2 were diluted with the blocking solution in a 5-fold gradient (100 µL added to 400 µL), respectively. The containment solution was discarded, 300 µL of PBS-T was washed 3 times, 100 µL of sdAb39 dilutions of different dilutions were added to each well, and 25 µL of ACE2 dilutions of different dilutions were added to the competition group at RT, the lowest rotation speed, for 60 min. The latter experimental steps are the same as for ELISA.

### Neutralization test of pseudovirus

To determine the neutralization activity of sdAb39, a pseudovirus neutralization assay was carried out, and this experiment was entrusted to Darui Biotechnology Co. The main materials included: Huh7 cells (JCRB, 00403), novel coronavirus (SARS-CoV-2) pseudoviral strains WH-1 (Darui, DR-XG-A001) and BA.2 (Darui, DR-XG-C011), DMEM high-sucrose medium (Gibco, C11995500BT), fetal bovine serum (ExCell Bio, FSP500), penicillin-streptomycin double antibody (Gibco, 15,140,163), and firefly luciferase detection reagent (DR-FLUC-03). This experimental procedure was done under BSL-2 biosafety level conditions.

In 96-well plates, SARS-Cov-2 pseudoviruses (500–1000 TCID50/well) were pre-incubated with different dilutions of sdAb39 or TY1 for 1 h at 37 °C, respectively. These virus-sdAb39 or virus-TY1mixtures were then added to Huh7/hACE2 cells in 96-well plates (10^4^ per well), respectively. Approximately 20–28 h after inoculation, the supernatant was aspirated and discarded, then 100 µL of luciferase detection reagent was added and the reaction was carried out for 2–5 min at room temperature and protected from light. Blow the liquid in the reaction wells 6–8 times with a multichannel pipette to fully lyse the cells. Transfer to the corresponding 96-well chemiluminescence detection plate, and put it into the multifunctional microtiter detector (PE Ensight) to read the luminescence value. The half-maximal inhibitory concentration (IC50) values were determined by the four-parameter nonlinear regression model of PRISM. All experiments were performed in triplicate and repeated at least twice.

### Biolayer interferometry (BLI)

Binding of sdAb39 and ACE2 to RBD was assessed using the Octet RED384 system (FortéBio). To determine the apparent affinity (Kd), 10ug/mL recombinant wild-type RBD-Fc (Sino Biological, 40,592-V02H) was loaded (~ 1.0 nm) onto Protein A biosensors (Molecular Devices, 18-5010). After a 100s baseline step in PBS, the RBD-Fc loaded biosensors were immersed in sdAb39 or ACE2s solution for a 200s association step, followed by a 200s dissociation step in PBS. All data for each sample were aligned, and Kd was determined with a 1:1 binding model in FortéBio data analysis software, version 12.1.

## Results

### Initial screening of anti-RBD sdAb

**Fig. 2:**
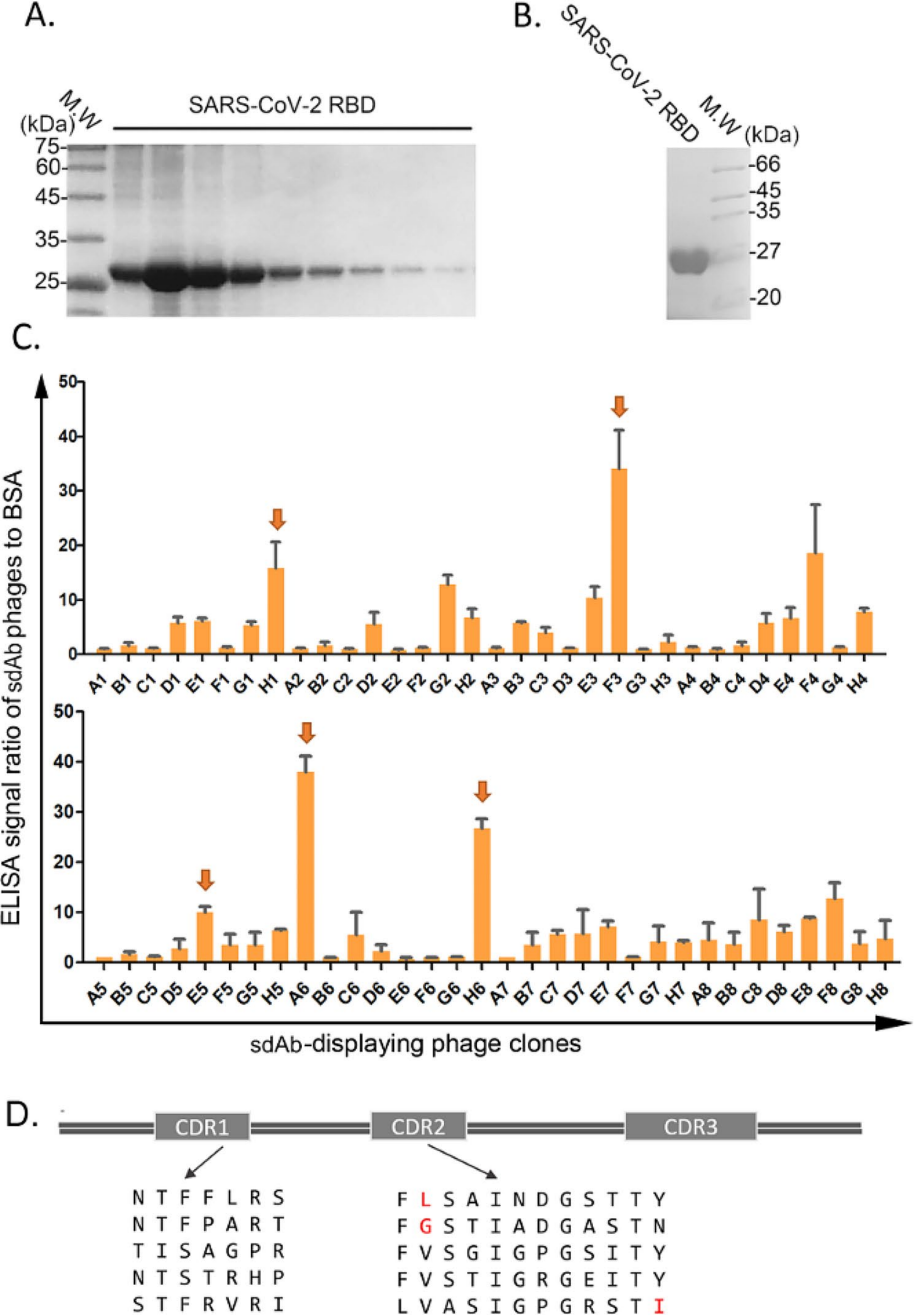
Initial screening of anti-RBD sdAbs. (**A-B**) Expression and purification of the recombinant RBD through baculovirus expression system and purification by Ni–NTA followed by gel filtration. C. The sequences of the CDR1 and CDR2 of the five high-affinity RBD-binding sdAbs. D. The results of phage ELISA of candidate phages. The error bars represent S.D. from three independent experiments. The statistical difference was measured by paired two-sided Student’s *t*-test

We obtained the recombinant SARS-CoV-2 S protein RBD (as shown in Fig. 2A-B) through the Bac-to-Bac baculovirus expression system and purified by Ni–NTA affinity column followed by Superdex-75 gel filtration column. Two rounds of phage panning were performed against the recombinant RBD from the sdAb-U library (Table 1). Enrichment of sdAb-displaying phages against the RBD was monitored by enzyme-linked immunosorbent assay (ELISA). High-affinity sdAbs were successfully enriched by these repeated (25–30 wash times) and stringent (0.5% Tween 20) elution procedures. Recovery efficiency was estimated by dividing the output phage titer by the input phage titer, as shown in Table 2. The recovery efficiency for each round was calculated by dividing the output titer by the input titer. Candidate phages enriched more than 5-fold over the bovine serum albumin (BSA) control protein were selected as initial leads. No negative controls other than BSA were used. Five RBD-binding sdAb clones (Fig. 2C) with high ELISA signal-to-background ratios (S/B) were sequenced.

**Table 1:** After sequential rounds of screening using the universal library sdAb-U and the targeted library sdAb-RBD, the SARS-CoV-2 RBD-specific binding phages were selectively enriched. PFU, plaque-forming unit

Library	Round	Wash times	Input titer (PFU/well)	Output titer (PFU/well)	Recovery efficiency	Fold increase
sdAb-U	Round 1	25	3.2 × 10^11^	1.2 × 10^6^	3.8 × 10^− 6^	--
	Round 2	30	2.1 × 10^10^	7.1 × 10^6^	3.4 × 10^− 4^	89
sdAb-RBD	Round 1	25	2.5 × 10^10^	3.9 × 10^6^	1.6 × 10^− 4^	42 (to sdAb-U round 1)
	Round 2	30	5.1 × 10^9^	1.0 × 10^6^	2.0 × 10^− 3^	13

**Table 2:** SARS-CoV-2 pseudovirus strains

Pseudovirus strain	Mutation site
WH-1	N/A
BA.2	T19I、L24S、P25del、P26del、A27del、G142D、V213G、G339D、S371F、S373P、S375F、T376A、D405N、R408S、K417N、N440K、S477N、T478K、E484A、Q493R、Q498R、N501Y、Y505H、D614G、H655Y、N679K、P681H、N764K、D796Y、Q954H、N969K

Their CDR1 sequences were: (1) NTFFLRS, (2) NTFPART, (3) TISAGPR, (4) NTSTRHP, (5) STFRVRI; and their CDR2 sequences were: (1) FLSAINDGSTTY, (2) FGSTIADGASTN, (3) FVSGIGPGSITY, (4) FVSTIGRGEITY, (5) LVASIGPGRSTI (Fig. 2D). These CDR1 and CDR2 sequences are consistent with the sequence characteristics of sdAbs (Fig. 2D). Then, we combined these CDR1s and CDR2s with a fully randomized CDR3 library (as in Fig. 1) to construct an RBD-specific sdAb library. We synthesized the five CDR1 fragments and the five CDR2 fragments, and randomly combined them (5 × 5 = 25 combinations) with the randomly mutated CDR3 library to construct a new library, sdAb-RBD (Fig. 2D). sdAb-RBD using the same methods as the construction of sdAb-U.

### Anti-RBD sdAb screening from the focused library

Based on the screening results of the universal library, the diversity of CDR1 and CDR2 was narrowed down. Two rounds of phage panning were performed with the sdAb-RBD targeting library. High-affinity sdAbs were successfully enriched by these repeated and stringent elution procedures. Recovery efficiency was estimated by dividing the output phage titer by the input phage titer, as shown in Table 1. The recovery efficiency of bound phage obtained from the targeted sdAb-RBD library was higher than that from the universal sdAb-U library by 42 times. The recovery efficiency of the second round of panning is higher than the first round of panning in either the initial library sdAb-U (by 89 times increased) or the subsequent focused library sdAb-RBD (by 13 times increased).

**Fig. 3:**
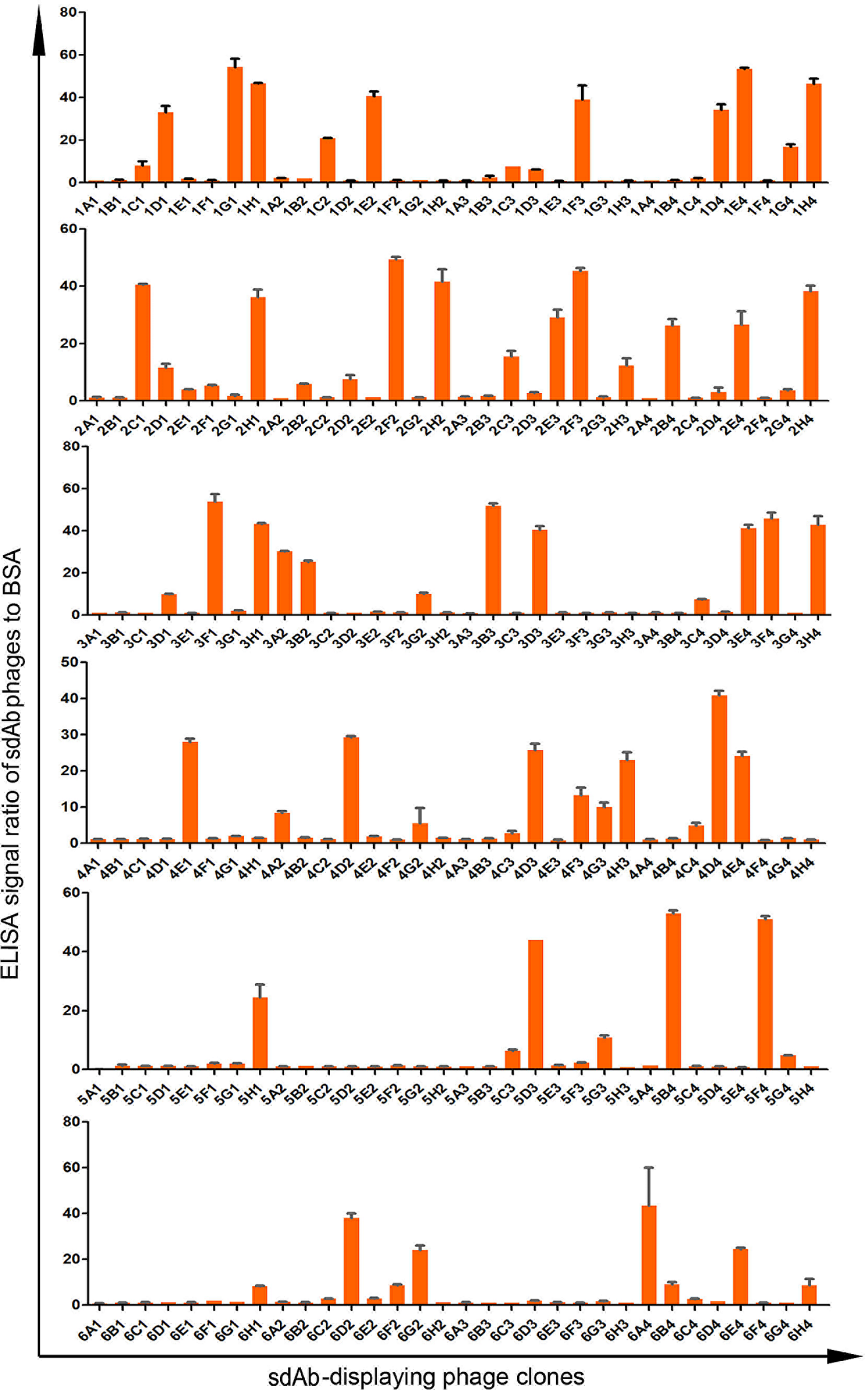
The results of phage ELISA of candidate phages. The error bars represent S.D. from three independent experiments

**Fig. 4:**
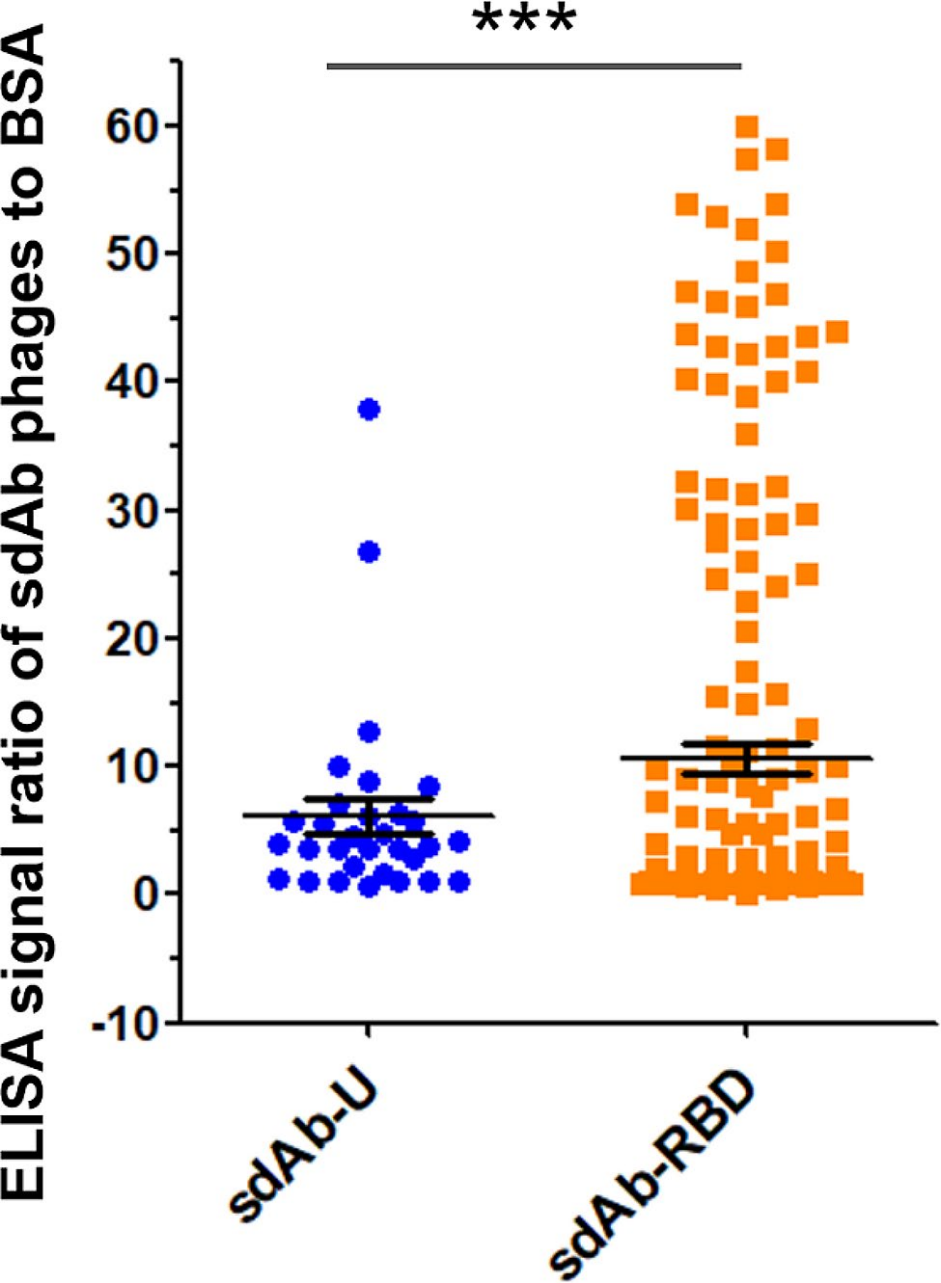
Affinity of sdAbs phage clones screened for SARS-CoV-2 RBD binding from the universal sdAb-U library or the target sdAb-RBD library. The statistical difference was measured by paired two-sided Student’s *t*-test (***, *P* < 0.001)

From the sdAb-RBD library, dozens of candidate sdAb-displaying phage clones with more than 30-fold enhanced ELISA signals over BSA were selected (Fig. 3). On average, the sdAb phage clones screened from the targeted sdAb-RBD library had a significantly stronger affinity to the RBD than those from the universal sdAb-U library (Fig. 4). These results showed that the sdAb-RBD library, with CDR1 and CDR2 optimized according to the lead sequences, had improved neutralizing capacity against RBD compared with the initial library sdAb-U. It’s worth noting that the presence of high-molecular weight aggregates of the sdAbs and RBD proteins was not assessed but was likely and could dramatically affect the results, and positive and negative controls other than BSA were not included in the titration ELISA, and therefore the binding specificity could not be evaluated.

### Binding affinity of the top sdAb candidates

**Fig. 5:**
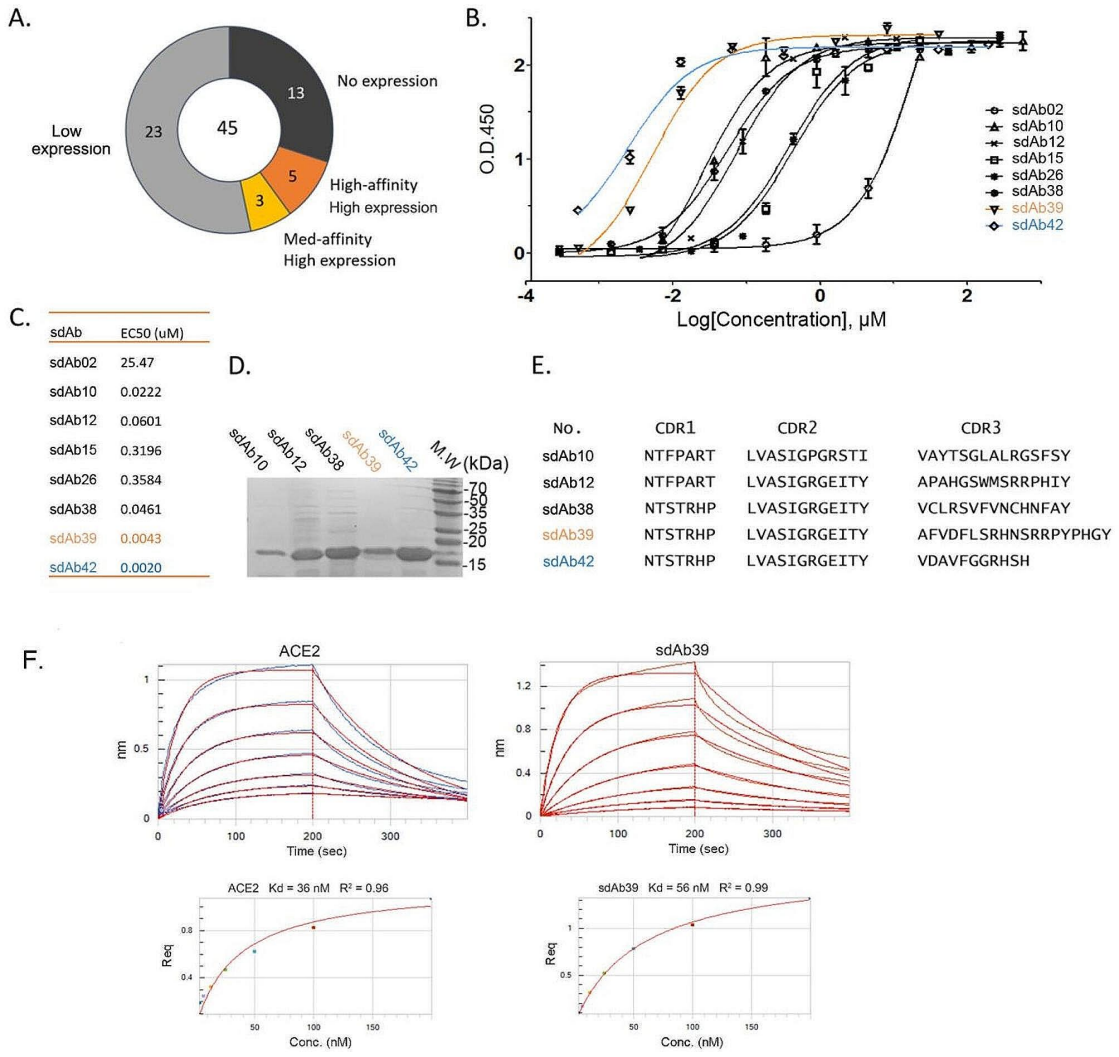
RBD binding affinity of the sdAb candidates. (**A**) Pie chart of RBD sdAbs’ affinity and expression in *E. coli*. High expression indicated more than 5 mg sdAbs protein could be purified from 1 L of *E. coli* broth. (**B**) RBD binding affinity analysis of the sdAb candidates by ELISA. (**C**) EC_50_ of the sdAb candidates binding to RBD. The EC_50_ is the concentration of sdAbs that gives a half-maximal RBD-binding response. (**D**) Five highest affinity RBD sdAb candidates. (**E**)` The amino acid sequence of the five highest affinity RBD sdAb candidates. (**F**) The binding affinity of sdAb39 and ACE2 to wild-type RBD. The upper panel: the association and dissociation of the response curves of each sample. Fc-tagged SARS-CoV-2 RBDs were loaded onto the surface of Protein A biosensors. A series of concentrations of each sample were used. The lower panel: Kd of each sample was fitted based on the experimental data

For *E. coli* expression of phage-screened sdAb, we selected 45 highest-affinity clones with diverse CDR sequences to encompass a variety of biophysical, structural, and potentially different antiviral properties. We found that 13 of them had no expression (28.8%), 23 had low expression (51%), and only 8 candidates had high expression (17.7%; High expression indicated more than 5 mg sdAbs protein could be purified from 1 L of *E. coli* broth) (Fig. 5A). Solubility of the proteins was not assessed but because of the nature of synthetic human sdAbs (with exposed hydrophobic FR2 regions), they may aggregate. We purified these 8 sdAbs and tested their RBD binding by ELISA, from which we identified five high-affinity RBD-specific sdAbs (Fig. 5B-E). Amino acid sequences of the five highest affinity RBD sdAbs candidates are shown in Fig. 5E. Among them, sdAb39 and sdAb42 had the highest affinity to RBD, however, the sdAb42 protein was less stable and precipitated after freezing and thawing. sdAb39 has good stability, a high affinity to RBD, and the half-inhibitory concentration reaches about 10 nM (Fig. 5B-E).

The affinity of the most promising binder sdAb39 against the wild-type RBD was further determined using BLI [45]. Consistent with the previous ELISA result, the apparent affinity (Kd, dissociation constants) of sdAb39 to RBD was 56 nM (Fig. 5F). By the same BLI method, we also measured the Kd of ACE2 binding to RBD to be 36 nM, which is consistent with previously reports [46]. sdAb39 is identified as a promising binder of w RBD by both ELISA and BLI.

### Candidate sdAb competes with ACE2 for RBD binding

**Fig. 6:**
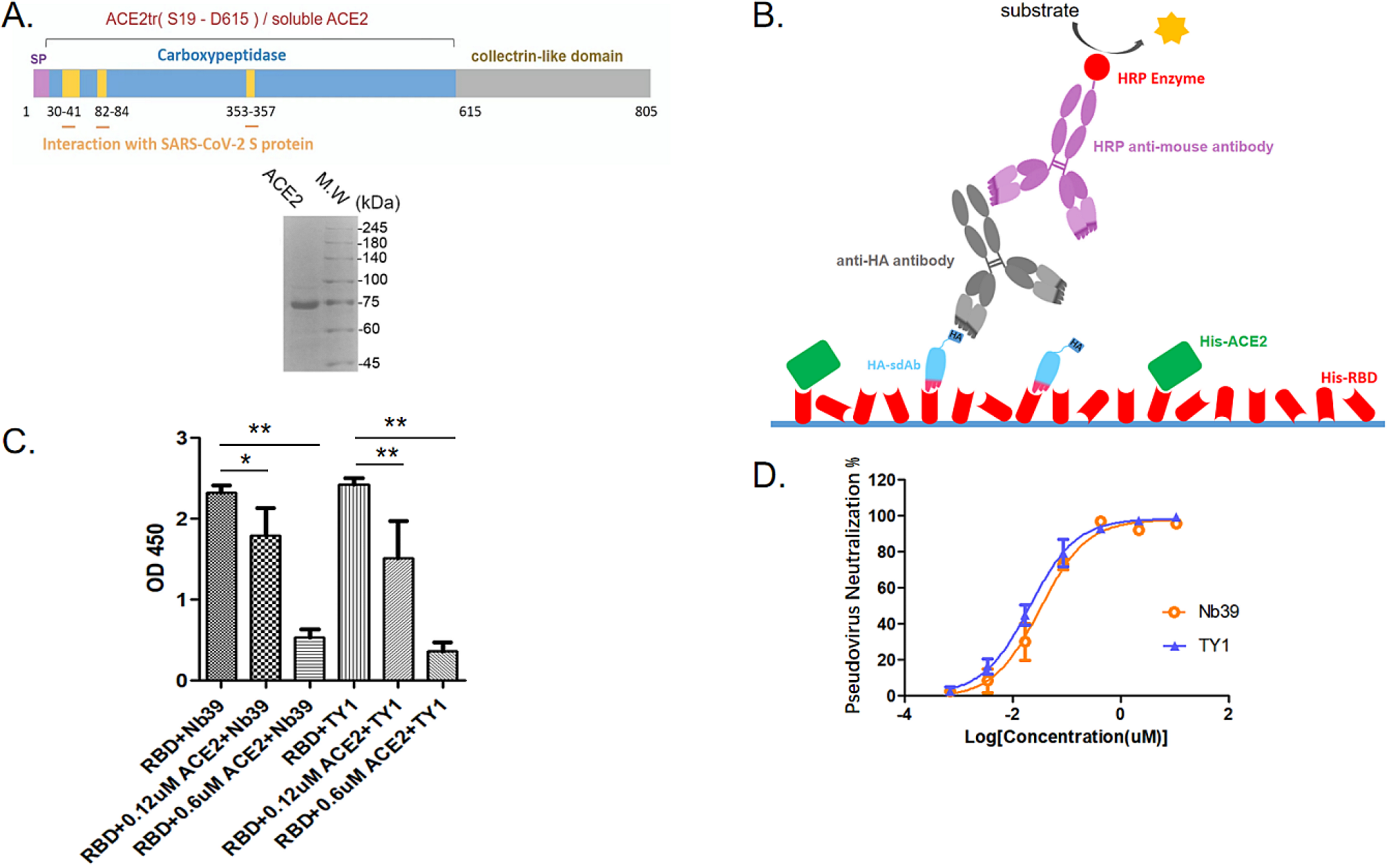
sdAb39 competes with ACE2 for RBD binding and neutralizes SARS-CoV-2 pseudoviruses. The statistical difference was measured by paired two-sided Student’s *t-*test (*, *P* < 0.05; **, *P* < 0.01)

To test whether the sdAb candidates compete with angiotensin-converting enzyme 2 (ACE2) for RBD binding. We obtained the extracellular domain of ACE2 protein through the Bac-to-Bac baculovirus expression system, and purified it by Ni–NTA affinity column followed by Superdex-75 gel filtration column (Fig. 6A). We also expressed and tested the SARS-CoV-2 nanobody TY1 [27], as a positive control. We developed a competition ELISA to test the competition between ACE2 and sdAb39 after mixing purified sdAb39 with SARS-CoV-2 RBD (Fig. 6B). The results showed that ACE2 could block the interaction between human sdAb39 and RBD (Fig. 6B-C); that is, ACE2 hindered the binding of RBD and sdAb39, allowing the no longer bound sdAb39 to be removed during plate washing. At a concentration of 0.6 µM, ACE2 showed a significant binding blocking effect, which remained significant at a concentration of 0.12 µM. Neither non-SARS-CoV-2 antibodies nor irrelevant competitors were tested in this study. The above experimental results indicated that sdAb39 blocked ACE2-RBD binding.

### **Neutralizing capacity of SARS-CoV-2 pseudoviruses for sdAb39**

The pseudoviruses neutralizing assay results showed that sdAb39 efficiently neutralized SARS-CoV-2 WH-1 pseudovirus with an IC_50_ value of 30 nM (Fig. 6D). However, using 30 nM sdAb39 we failed to observe significant omicron BA.2 pseudovirus neutralization. This is consistent with many studies concluding that the SARS-CoV-2 omicron mutant escapes most existing NAbs (Neutralizing antibodies) [10, 11, 47]. Non-SARS-CoV-2 pseudotyped viruses were not tested. These results suggest that the combined use of sdAb-U and sdAb-RBD libraries can lead to functional RBD antibodies.

## Discussion

The synthetic sdAb library uses gene synthesis technology to introduce random DNA sequences at specific sites, which is highly controllable and allows fast and efficient screening against target proteins. However, due to combinatorial explosion, the possible sdAb sequence space is extremely vast and much larger than the capacity of the phage library. In this study, we first screened universal sdAb-U library against the target protein and obtained five high-affinity lead sequences. We further assembled the CDR1 and CDR2 sequences from these five initial leads with randomly mutated CDR3 to construct a second library sdAb-RBD, which is more focused on the target protein (RBD). Multiple sdAb candidates were obtained from the sdAb-RBD library. Among them, sdAb39 has good stability, and high binding affinity to RBD with the EC_50_ reaching 4nM, and sdAb39 can compete with ACE2 for binding to RBD.

It’s worth noting that, the CDR3 amino acid sequences of the top five candidate SARS-CoV-2 RBD sdAbs (Fig. 5E) with the highest affinity display an intriguing pattern. All five sdAbs exhibit the presence of Arg amino acids within their CDR3 regions. Specifically, sdAb10, sdAb38, and sdAb42 contain a single Arg amino acid, while sdAb12 possesses two Arg amino acids, and sdAb39 incorporates three Arg amino acids. Furthermore, four out of the five sdAbs feature His amino acids, being found in sdAb12, sdAb38, sdAb39, and sdAb42. Notably, both sdAb12 and sdAb39 also contain an additional two Pro amino acids each. Additionally, sdAb12 incorporates one Met amino acid, whereas sdAb38 encompasses two Cys amino acids.

Studies have revealed the determinants of polyreactivity in antibodies, including CDR3 length and flexibility, hydrophobicity, net charge, as well as enrichment in Arg, Trp, Tyr residues [48–50]. In our study, all of the five selected sdAbs have at least one Arg, and sdAb12 incorporates one Met amino acid. Antibodies with positively charged CDRs (especially Arg) have a higher risk of low specificity than antibodies with negatively charged CDRs [51]. However, extensive studies have found that no single amino acid residue or biochemical property leads to polyreactivity, and more data support the use of multiple strategies for antibodies’ polyreactive binding. Therefore, we predicted the degree of polyreactivity of the RBD-binding sdAbs using the machine learning models developed by Harvey et al. (http://18.224.60.30:3000/) [50]. The predicted scores ( Fig. S1 and Table S1) of the five SARS-CoV-2 RBD sdAbs in Fig. 5E are within the range of minimal polyreactivity, except sdAb12. Interestingly, both sdAb39 with three Arg residues and sdAb42 with only one Arg residue in CDR3 have quite high scores (low polyreactivity). While Arg and unpaired Met residues may be potential factors contributing to the non-specific binding of proteins, however, both local and global characteristics of antibodies affect their degree of polyreactivity.

Arginine (Arg) is frequently involved in protein interactions due to its unique properties. The positive charge on its side chain enables strong electrostatic interactions, making it prone to engage in protein binding. Additionally, the aromatic ring of Arg can undergo π-interactions, further contributing to ligand binding. In terms of protein-protein interaction (PPI) hotspot residues, Tryptophan (21%), arginine (13.3%), and tyrosine (12.3%) have been found to emerge as the three fundamental amino acids with notable frequencies [52]. Arg has also been observed in the CDR sequences of nanobodies targeting SARS-CoV-2 RBD in published literature, such as the WNb7, WNb10, and WNb15 nanobodies [28].

**Fig. 7:**
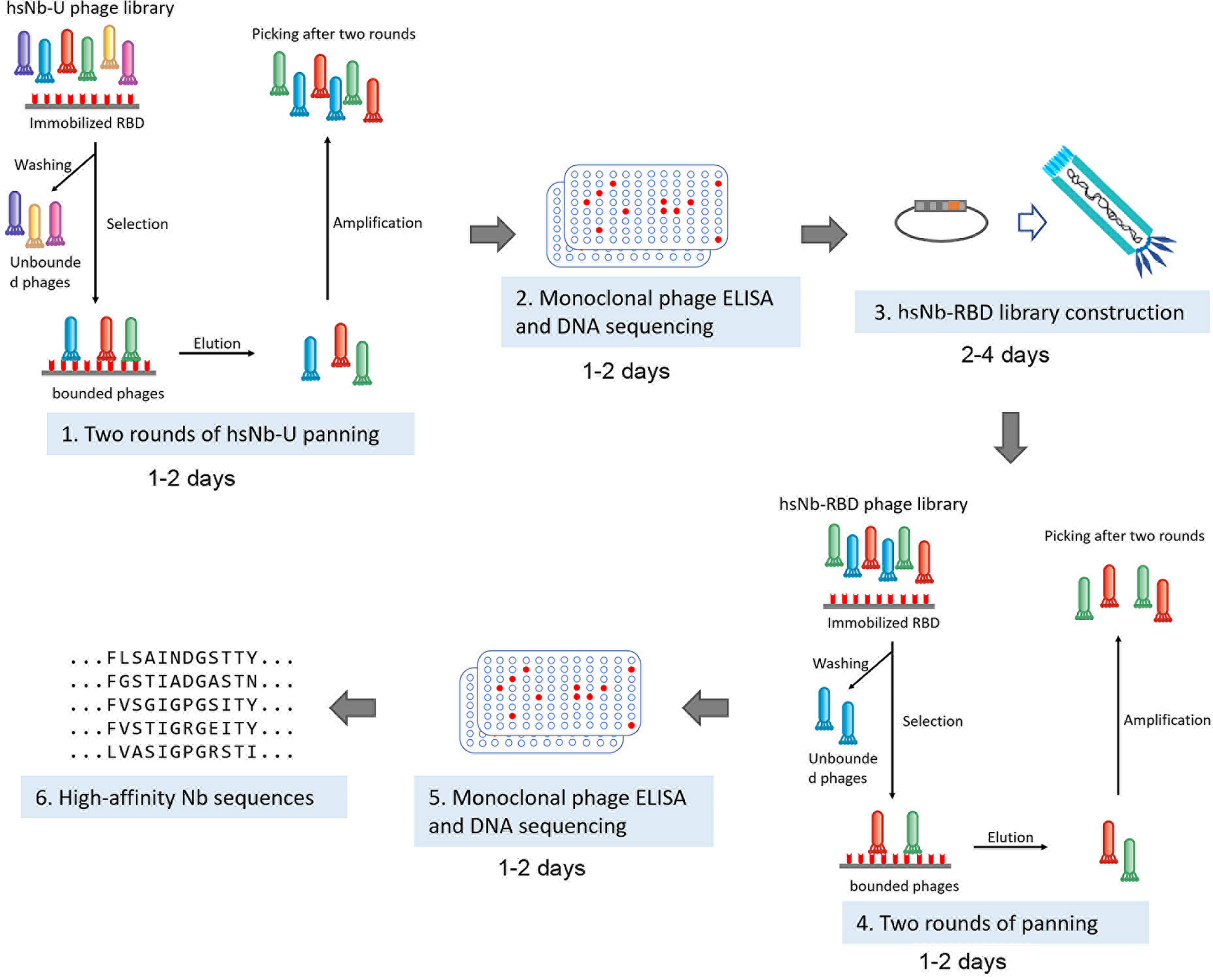
Strategy and timeline of the two-stage sdAb phage screening

The probability of screening high-affinity RBD-binding sdAb from the focused sdAb-RBD library is higher than from the universal sdAb library, and the identified sdAbs have higher affinities than the lead sequences. These results validate the feasibility and effectiveness of this two-stage screening strategy, where a universal and diverse library is initially screened to obtain lead sequences for construction of an antigen-specific library for a second-stage screening (Fig. 7). This strategy allows screening of high affinity SARS-CoV-2 sdAbs for about two weeks, which is relatively quickly compared to immunized animals. Moreover, compared to the traditional one-stage synthetic library screening (universal library in the first stage), the second-stage focused library is more efficient in screening. This strategy can be extended to the screening of other targets.

However, in vitro screening methods for phage synthetic libraries, selection is usually performed using antigens adsorbed directly onto the surface of the immunotubes, and non-specific adsorption may result in antigen denaturation. (1) Folding Stability and Solubility Problems: Since sequences in synthetic libraries are usually not subjected to evolutionary screening, there may be problems with folding stability and solubility. This may limit the application value of some candidates. (2) Risk of specificity and affinity: single-domain antibodies in synthetic libraries may show lower specificity and affinity, partly due to the absence of an evolutionary process of natural selection. (3) Design and construction constraints: the construction of synthetic libraries requires a full understanding and tuning of the interactions between the antigen and the structure of the sdAb.

In summary, here we report a synthetic sdAb platform for rapid selection of anti-RBD sdAb (Fig. 7), and this pipeline can be extended to the panning of other targets. These sdAb may be promising candidates for COVID-19 prevention, treatment, or as reagents to facilitate SARS-CoV-2 vaccine development. This two-stage strategy can be used to rapidly develop new sdAb against mutant virus strains, and address the need for continuous virus mutation in a pandemic. It’s worth noting that our sdAb phage synthetic libraries and strategy offer the advantages of high throughput, ease of use, and versatility, and can be used to obtain targeted binders rapidly and cost-effectively. However, fold stability and solubility issues as well as specificity and affinity challenges still needs to be addressed. Researchers need to consider the applicability of synthetic libraries based on specific goals and applications, and design and optimize them as needed. Besides, the short half-life of sdAb in circulation need to be addressed before sdAb can be considered for in vivo application. A very effective approach is to fuse sdAb with the human IgG1 Fc, which significantly extends their half-life.

### Electronic supplementary material

Below is the link to the electronic supplementary material.

Supplementary Material 1

Supplementary Material 2

## Data Availability

The sequences of primers and proteins of this study are presented in the supplementary Excel file. The other data and materials supporting the conclusions of the study are available from the corresponding author upon reasonable request.

## Abbreviations

COVID-19: Coronavirus disease 2019
SARS-CoV-2: Severe acute respiratory syndrome coronavirus 2
Nbs: Nanobodies
sdAb-U: Human synthetic single-domain antibody library - Universal
RBD: Receptor-binding domain
CDRs: Complementary determine regions
mAb: Monoclonal antibodies
WHO: World Health Organization
VOC: Variants of Concern
VHHs: Variable domains of heavy-chain–only antibodies
sdAb: Single-domains antibody
ELISA: Enzyme-linked immunosorbent assay
BSA: Bovine serum albumin
ACE2: Angiotensin-converting enzyme 2

## References

[1] Zhu N, Zhang D, Wang W, Li X, Yang B, Song J, Zhao X, Huang B, Shi W, Lu R (2020). A novel coronavirus from patients with Pneumonia in China, 2019. N Engl J Med.

[2] Zhou P, Yang XL, Wang XG, Hu B, Zhang L, Zhang W, Si HR, Zhu Y, Li B, Huang CL (2020). A pneumonia outbreak associated with a new coronavirus of probable bat origin. Nature.

[3] Abraham J (2020). Passive antibody therapy in COVID-19. Nat Rev Immunol.

[4] Zost SJ, Gilchuk P, Case JB, Binshtein E, Chen RE, Nkolola JP, Schafer A, Reidy JX, Trivette A, Nargi RS (2020). Potently neutralizing and protective human antibodies against SARS-CoV-2. Nature.

[5] Chen P, Nirula A, Heller B, Gottlieb RL, Boscia J, Morris J, Huhn G, Cardona J, Mocherla B, Stosor V (2021). SARS-CoV-2 neutralizing antibody LY-CoV555 in outpatients with Covid-19. N Engl J Med.

[6] Hansen J, Baum A, Pascal KE, Russo V, Giordano S, Wloga E, Fulton BO, Yan Y, Koon K, Patel K (2020). Studies in humanized mice and convalescent humans yield a SARS-CoV-2 antibody cocktail. Science.

[7] Shi R, Shan C, Duan X, Chen Z, Liu P, Song J, Song T, Bi X, Han C, Wu L (2020). A human neutralizing antibody targets the receptor-binding site of SARS-CoV-2. Nature.

[8] Ju B, Zhang Q, Ge J, Wang R, Sun J, Ge X, Yu J, Shan S, Zhou B, Song S (2020). Human neutralizing antibodies elicited by SARS-CoV-2 infection. Nature.

[9] Iketani S, Liu L, Guo Y, Liu L, Chan JF, Huang Y, Wang M, Luo Y, Yu J, Chu H (2022). Antibody evasion properties of SARS-CoV-2 Omicron sublineages. Nature.

[10] Planas D, Saunders N, Maes P, Guivel-Benhassine F, Planchais C, Buchrieser J, Bolland WH, Porrot F, Staropoli I, Lemoine F (2022). Considerable escape of SARS-CoV-2 Omicron to antibody neutralization. Nature.

[11] Liu L, Iketani S, Guo Y, Chan JF, Wang M, Liu L, Luo Y, Chu H, Huang Y, Nair MS (2022). Striking antibody evasion manifested by the Omicron variant of SARS-CoV-2. Nature.

[12] Fernandes Q, Inchakalody VP, Merhi M, Mestiri S, Taib N, Moustafa Abo El-Ella D, Bedhiafi T, Raza A, Al-Zaidan L, Mohsen MO (2022). Emerging COVID-19 variants and their impact on SARS-CoV-2 diagnosis, therapeutics and vaccines. Ann Med.

[13] Muyldermans S (2013). Nanobodies: natural single-domain antibodies. Annu Rev Biochem.

[14] Hamers-Casterman C, Atarhouch T, Muyldermans S, Robinson G, Hamers C, Songa EB, Bendahman N, Hamers R (1993). Naturally occurring antibodies devoid of light chains. Nature.

[15] Nguyen VK, Hamers R, Wyns L, Muyldermans S (2000). Camel heavy-chain antibodies: diverse germline V(H)H and specific mechanisms enlarge the antigen-binding repertoire. EMBO J.

[16] Rasmussen SG, Choi HJ, Fung JJ, Pardon E, Casarosa P, Chae PS, Devree BT, Rosenbaum DM, Thian FS, Kobilka TS (2011). Structure of a nanobody-stabilized active state of the beta(2) adrenoceptor. Nature.

[17] Bathula NV, Bommadevara H, Hayes JM (2021). Nanobodies: the future of antibody-based Immune therapeutics. Cancer Biother Radiopharm.

[18] Steeland S, Vandenbroucke RE, Libert C (2016). Nanobodies as therapeutics: big opportunities for small antibodies. Drug Discovery Today.

[19] Wu Y, Jiang S, Ying T (2017). Single-domain antibodies as therapeutics against human viral diseases. Front Immunol.

[20] Duggan S (2018). Caplacizumab: First Global approval. Drugs.

[21] Bruce VJ, McNaughton BR (2017). Evaluation of Nanobody conjugates and protein fusions as Bioanalytical reagents. Anal Chem.

[22] Desmyter A, Spinelli S, Roussel A, Cambillau C (2015). Camelid nanobodies: killing two birds with one stone. Curr Opin Struct Biol.

[23] Vincke C, Loris R, Saerens D, Martinez-Rodriguez S, Muyldermans S, Conrath K (2009). General strategy to humanize a camelid single-domain antibody and identification of a universal humanized nanobody scaffold. J Biol Chem.

[24] Moutel S, Bery N, Bernard V, Keller L, Lemesre E, de Marco A, Ligat L, Rain JC, Favre G, Olichon A et al. NaLi-H1: a universal synthetic library of humanized nanobodies providing highly functional antibodies and intrabodies. eLife 2016, 5. 10.7554/eLife.16228 PMC4985285 27434673

[25] Wu Y, Li C, Xia S, Tian X, Kong Y, Wang Z, Gu C, Zhang R, Tu C, Xie Y (2020). Identification of human single-domain antibodies against SARS-CoV-2. Cell Host Microbe.

[26] Fan X, Cao D, Kong L, Zhang X (2020). Cryo-EM analysis of the post-fusion structure of the SARS-CoV spike glycoprotein. Nat Commun.

[27] Hanke L, Vidakovics Perez L, Sheward DJ, Das H, Schulte T, Moliner-Morro A, Corcoran M, Achour A, Karlsson Hedestam GB, Hallberg BM (2020). An alpaca nanobody neutralizes SARS-CoV-2 by blocking receptor interaction. Nat Commun.

[28] Pymm P, Adair A, Chan LJ, Cooney JP, Mordant FL, Allison CC, Lopez E, Haycroft ER, O’Neill MT, Tan LL et al. Nanobody cocktails potently neutralize SARS-CoV-2 D614G N501Y variant and protect mice. *Proceedings of the National Academy of Sciences of the United States of America* 2021, 118(19). 10.1073/pnas.2101918118 PMC8126837 33893175

[29] Nambulli S, Xiang Y, Tilston-Lunel NL, Rennick LJ, Sang Z, Klimstra WB, Reed DS, Crossland NA, Shi Y, Duprex WP. Inhalable nanobody (PiN-21) prevents and treats SARS-CoV-2 infections in Syrian hamsters at ultra-low doses. Sci Adv 2021, 7(22). 10.1126/sciadv.abh0319 PMC8153718 34039613

[30] Chen F, Liu Z, Jiang F (2021). Prospects of neutralizing Nanobodies against SARS-CoV-2. Front Immunol.

[31] Koenig PA, Das H, Liu H, Kummerer BM, Gohr FN, Jenster LM, Schiffelers LDJ, Tesfamariam YM, Uchima M, Wuerth JD et al. Structure-guided multivalent nanobodies block SARS-CoV-2 infection and suppress mutational escape. *Science* 2021, 371(6530). 10.1126/science.abe6230 PMC7932109 33436526

[32] Xiang Y, Nambulli S, Xiao Z, Liu H, Sang Z, Duprex WP, Schneidman-Duhovny D, Zhang C, Shi Y (2020). Versatile and multivalent nanobodies efficiently neutralize SARS-CoV-2. Science.

[33] Gai J, Ma L, Li G, Zhu M, Qiao P, Li X, Zhang H, Zhang Y, Chen Y, Ji W (2021). A potent neutralizing nanobody against SARS-CoV-2 with inhaled delivery potential. MedComm (2020).

[34] Wrapp D, De Vlieger D, Corbett KS, Torres GM, Wang N, Van Breedam W, Roose K, van Schie L, Hoffmann M, Team V-CC-R (2020). Structural basis for potent neutralization of Betacoronaviruses by single-domain camelid antibodies. Cell.

[35] Smith GP, Petrenko VA (1997). Phage Display. Chem Rev.

[36] Winter G, Griffiths AD, Hawkins RE, Hoogenboom HR (1994). Making antibodies by phage display technology. Annu Rev Immunol.

[37] McMahon C, Baier AS, Pascolutti R, Wegrecki M, Zheng S, Ong JX, Erlandson SC, Hilger D, Rasmussen SGF, Ring AM (2018). Yeast surface display platform for rapid discovery of conformationally selective nanobodies. Nat Struct Mol Biol.

[38] Yan J, Li G, Hu Y, Ou W, Wan Y (2014). Construction of a synthetic phage-displayed Nanobody library with CDR3 regions randomized by trinucleotide cassettes for diagnostic applications. J Transl Med.

[39] Mitchell LS, Colwell LJ (2018). Comparative analysis of nanobody sequence and structure data. Proteins.

[40] Jacobs TM, Yumerefendi H, Kuhlman B, Leaver-Fay A (2015). SwiftLib: rapid degenerate-codon-library optimization through dynamic programming. Nucleic Acids Res.

[41] Young L, Dong Q (2004). Two-step total gene synthesis method. Nucleic Acids Res.

[42] Quan J, Tian J (2009). Circular polymerase extension cloning of complex gene libraries and pathways. PLoS ONE.

[43] Quan J, Tian J (2011). Circular polymerase extension cloning for high-throughput cloning of complex and combinatorial DNA libraries. Nat Protoc.

[44] Xiong AS, Yao QH, Peng RH, Duan H, Li X, Fan HQ, Cheng ZM, Li Y (2006). PCR-based accurate synthesis of long DNA sequences. Nat Protoc.

[45] Liu Y, Wang Z, Zhuang X, Zhang S, Chen Z, Zou Y, Sheng J, Li T, Tai W, Yu J (2023). Inactivated vaccine-elicited potent antibodies can broadly neutralize SARS-CoV-2 circulating variants. Nat Commun.

[46] Bayarri-Olmos R, Jarlhelt I, Johnsen LB, Hansen CB, Helgstrand C, Rose Bjelke J, Matthiesen F, Nielsen SD, Iversen KK, Ostrowski SR (2021). Functional effects of receptor-binding domain mutations of SARS-CoV-2 B.1.351 and P.1 variants. Front Immunol.

[47] Cao Y, Wang J, Jian F, Xiao T, Song W, Yisimayi A, Huang W, Li Q, Wang P, An R (2022). Omicron escapes the majority of existing SARS-CoV-2 neutralizing antibodies. Nature.

[48] Borowska MT, Boughter CT, Bunker JJ, Guthmiller JJ, Wilson PC, Roux B, Bendelac A, Adams EJ (2023). Biochemical and biophysical characterization of natural polyreactivity in antibodies. Cell Rep.

[49] Boughter CT, Borowska MT, Guthmiller JJ, Bendelac A, Wilson PC, Roux B, Adams EJ. Biochemical patterns of antibody polyreactivity revealed through a bioinformatics-based analysis of CDR loops. eLife 2020, 9. 10.7554/eLife.61393 PMC7755423 33169668

[50] Harvey EP, Shin JE, Skiba MA, Nemeth GR, Hurley JD, Wellner A, Shaw AY, Miranda VG, Min JK, Liu CC (2022). An in silico method to assess antibody fragment polyreactivity. Nat Commun.

[51] Rabia LA, Zhang Y, Ludwig SD, Julian MC, Tessier PM (2018). Net charge of antibody complementarity-determining regions is a key predictor of specificity. Protein Eng Des Sel.

[52] Morrow JK, Zhang S (2012). Computational prediction of protein hot spot residues. Curr Pharm Des.

