# Supplementary material for: Single-domain antibodies against SARS-CoV-2 RBD from a two-stage phage screening of universal and focused synthetic libraries
Source: BMC Infect Dis. 2024 Feb 13;24:199. doi: 10.1186/s12879-024-09022-8 (PMC10865538; doi:10.1186/s12879-024-09022-8)
Supplement: Supplementary file 2 — Supplementary Material 2 [file 12879_2024_9022_MOESM2_ESM.pdf]

Supplementary Information

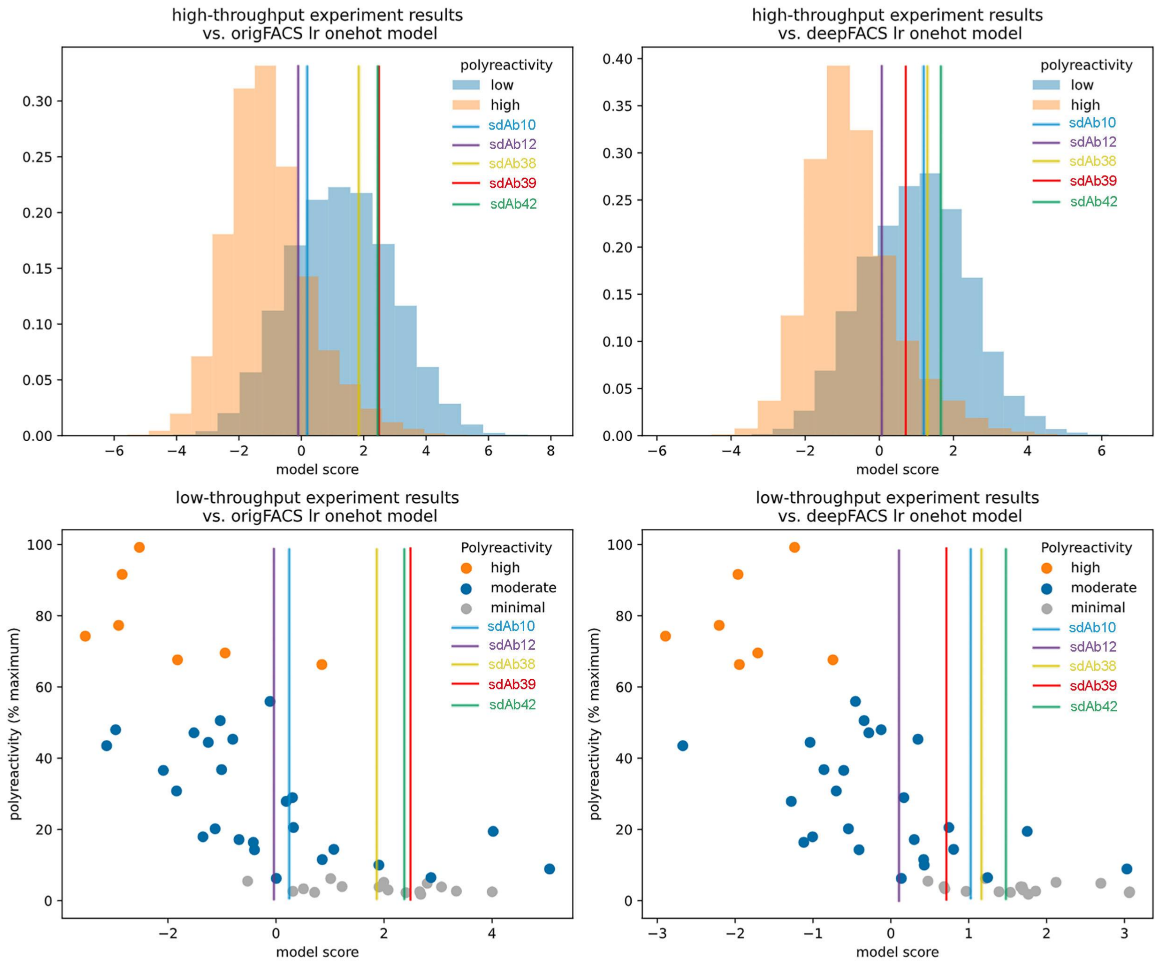

Figure S1. The predicted polyreactivity of the SARS-CoV-2 RBD sdAbs. A high score indicates low predicted polyreactivity, whereas a low score indicates increased polyreactivity.

Table S1. The model scores of the SARS-CoV-2 RBD sdAbs

ID	CDR1	CDR2	CDR3	Model score	
				origFACS lr onehot model	deepFACS lr onehot model
sdAb10	NTFPART	LVASIGPGRST	VAYTSGLALRGSFSY	0.24	1.03
sdAb12	NTFPART	LVASIGRGEIT	APAHGSWMSRRPHIY	-0.13	0.06
sdAb38	NTSTRHP	LVASIGRGEIT	VCLRSVFVNCHNFAY	1.89	1.13
sdAb39	NTSTRHP	LVASIGRGEIT	AFVDFLSRHNSRRPYPHGY	2.49	0.71
sdAb42	NTSTRHP	LVASIGRGEIT	VDAVFGGRHSH	2.41	1.43

Figure 2A

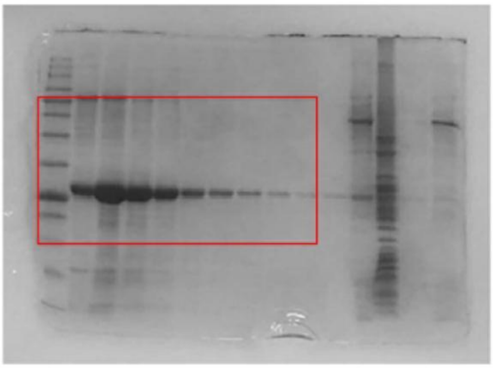

Figure 2B

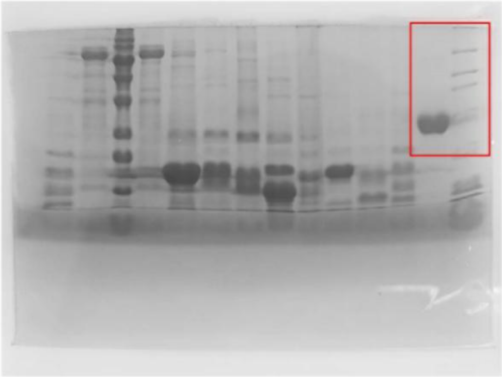

Figure 5D

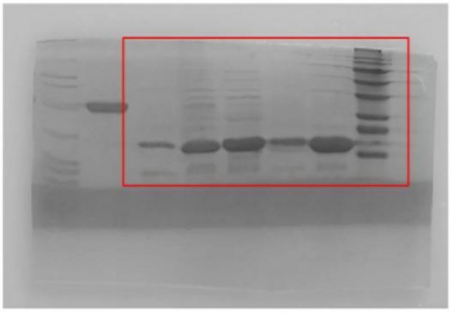

Figure 6A

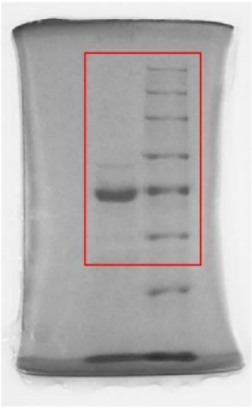

Figure S2. Uncropped gel images of Fig 2A/B, 5D, and 6A.
